# A new species and new records of *Onchidium* slugs (Gastropoda, Euthyneura, Pulmonata, Onchidiidae) in South-East Asia

**DOI:** 10.3897/zookeys.892.39524

**Published:** 2019-11-27

**Authors:** Benoît Dayrat, Tricia C. Goulding, Munawar Khalil, Deepak Apte, Shau Hwai Tan

**Affiliations:** 1 Pennsylvania State University, Department of Biology, Mueller Laboratory 514, University Park, PA 16802, USA; 2 Current address: Bernice Pauahi Bishop Museum, 1525 Bernice St, Honolulu, HI 96817, USA; 3 Department of Marine Science, Universitas Malikussaleh, Reuleut Main Campus, Kecamatan Muara Batu, North Aceh, Aceh, 24355, Indonesia; 4 Bombay Natural History Society, Hornbill House, Opp. Lion Gate, Shaheed Bhagat Singh Road, Mumbai 400 001, Maharashtra, India; 5 Marine Science Laboratory, School of Biological Sciences, Universiti Sains Malaysia, 11800 Minden Penang, Malaysia; 6 Centre for Marine and Coastal Studies, Universiti Sains Malaysia, 11800 Minden Penang, Malaysia

**Keywords:** Biodiversity, integrative taxonomy, Malacca Strait, mangrove, systematic revisions

## Abstract

A new species, *Onchidium melakense* Dayrat & Goulding, **sp. nov.**, is described, bringing the total to four known species in the genus *Onchidium* Buchannan, 1800. *Onchidium melakense* is a rare species with only nine individuals found at three mangrove sites in the Andaman Islands and the Strait of Malacca (western Peninsular Malaysia and eastern Sumatra). The new species is delineated based on mitochondrial (COI and 16S) and nuclear (ITS2 and 28S) DNA sequences as well as comparative anatomy. Each *Onchidium* species is characterized by a distinct color and can easily be identified in the field, even in the Strait of Malacca where there are three sympatric *Onchidium* species. An identification key is provided. In addition, *Onchidium stuxbergi* (Westerlund, 1883) is recorded for the first time from eastern Sumatra, and *Onchidium pallidipes* Tapparone-Canefri, 1889, of which the type material is described and illustrated here, is regarded as a new junior synonym of *O. stuxbergi*.

## Introduction

Onchidiids are true slugs (lacking an internal shell) which breathe air with a lung and die if they are immersed in water for a few hours. Most species are found in the intertidal zone, but a few species are adapted to high-elevation rainforest up to 1,850 meters (Dayrat 2010). Onchidiids are found worldwide, but the highest species diversity is in South-East Asia, especially in mangroves where onchidiids are among the most abundant animals. Onchidiid slugs are most closely related to veronicellids, which are also true slugs but which, unlike onchidiids, are fully terrestrial, and to Stylommatophora, the land snails and slugs (Dayrat et al. 2011).

The taxonomy of the Onchidiidae has been in a state of chaos for many years (Dayrat 2009). In the past few years, the Dayrat laboratory has been revising the taxonomy of the entire family one clade at a time (Dayrat et al. 2016, 2017, 2018, 2019a, b; Dayrat and Goulding 2017; Goulding et al. 2018a, b, c). We follow an integrative approach to taxonomy based on: 1) a re-examination of all types available and a comprehensive review of the nomenclature to address the application of all existing species- and genus-group names; 2) extensive field work to observe species in their habitat and to collect fresh material (we visited more than 300 sites worldwide); and, 3) species delineation using DNA sequences to complement comparative anatomy.

As the type genus of the family, *Onchidium* Buchannan, 1800 was the focus of our first revision (Dayrat et al. 2016). In the past, the generic name *Onchidium* was traditionally used by default for many onchidiid species, because relationships among onchidiid species were very confusing. In total, 80 species were described using *Onchidium* in the original binomial (Dayrat 2009). However, the revision of the genus *Onchidium* showed that it included only three species (Dayrat et al. 2016): the type species *O. typhae* Buchannan, 1800, *O. reevesii* (J. E. Gray, 1850), and *O. stuxbergi* (Westerlund, 1883). Except for *Onchidium nigrum* Plate, 1893 and *Onchidium pallidipes* Tapparone-Canefri, 1889, both junior synonyms of *O. stuxbergi*, all other species binomials with *Onchidium* as a generic name refer to species that actually belong to other onchidiid genera or to nomina dubia which cannot be placed in any onchidiid genus (Dayrat et al. 2016, 2017, 2018, 2019a, b; Dayrat and Goulding 2017; Goulding et al. 2018a, b, c).

*Onchidium* slugs can be identified in the field thanks to two external features: large, conical, pointed papillae on the dorsal notum and very long and thin ocular tentacles. These two traits are synapomorphies of *Onchidium* which are not found in any other onchidiids. In the present contribution, we describe the new species *Onchidium melakense* Dayrat & Goulding. It is a rare species for which we found only nine individuals at three mangrove sites (out of the dozens of sites that we explored in the region). One individual was collected in the Andaman Islands. Eight individuals were collected in the Strait of Malacca: four individuals in the Matang mangrove near Kuala Sepatang in western Peninsular Malaysia, and four individuals in Sinaboi Island, a small uninhabited island in eastern Sumatra. *Onchidium melakense* is supported by mitochondrial (COI and 16S) and nuclear (ITS2 and 28S) DNA sequences and comparative anatomy. Each *Onchidium* species is characterized by a distinct color so the three *Onchidium* species that are sympatric in the Strait of Malacca can easily be identified: *Onchidium melakense* is characterized by a light brown dorsal notum and a perfectly white hyponotum. An identification key to *Onchidium* species is provided.

In addition, *Onchidium stuxbergi* is recorded for the first time from eastern Sumatra. Also, *Onchidium pallidipes* Tapparone-Canefri, 1889, of which the type material is described and illustrated here, is regarded as a new junior synonym of *O. stuxbergi*. Finally, for the first time, a plate illustrates precisely the range of individual variation for the intestinal loops of each *Onchidium* species: intestinal loops are of type II in *O. typhae* and of type III in the three other species.

## Materials and methods

### Collecting

All specimens were collected by the authors in the past few years, except six specimens from China for which sequences were obtained from GenBank (Table 1). Collecting field parties were led by Benoît Dayrat in the Andaman Islands (India) and Peninsular Malaysia and by Munawar Khalil in Sumatra (Indonesia). Sites were accessed by car or by boat. Although each site was explored for an average of two hours, the exact time spent at each site also depended on the time of the low tide, the weather conditions, etc. Photographs were taken to document the kind of mangrove being visited as well as the diverse microhabitats where specimens were collected.

**Table 1.** DNA extraction numbers and GenBank accession numbers for all the specimens included in the present study. The letter H next to an extraction number indicates the holotype.

Species	DNA #	Voucher	Locality	COI	16S	ITS2	28S
*Onchidium melakense*	1105	BNHS 94	Andaman, India	–	MN528066	–	–
	1720	UMIZ 00001	Sumatra, Indonesia	MN528057	MN528067	MN527565	MN527530
	1723	UMIZ 00001	Sumatra, Indonesia	MN528058	MN528068	–	–
	1769	UMIZ 00001	Sumatra, Indonesia	MN528059	MN528069	MN527566	MN527531
	1771	UMIZ 00001	Sumatra, Indonesia	MN528060	MN528070	MN527567	MN527532
	5978	USMMC 00076	Peninsular Malaysia	MN528061	MN528071	–	–
	5979 H	USMMC 00075	Peninsular Malaysia	MN528062	MN528072	MN527568	MN527533
	5981	USMMC 00076	Peninsular Malaysia	MN528063	MN528073	MN527569	MN527534
	5982	USMMC 00076	Peninsular Malaysia	MN528064	MN528074	–	–
*O. reevesii*	S871	ASTM-Mo-S871	China (22°30’N)	JN543161*	JN543097*	–	–
	S831	ASTM-Mo-S831	China (24°24’N)	JN543160*	JN543096*	–	–
	S853	ASTM-Mo-S853	China (27°29’N)	JN543164*	JN543100*	–	–
	S821	ASTM-Mo-S821	China (33°20’N)	JN543162*	JN543098*	–	–
	S802	ASTM-Mo-S802	China (34°46’N)	JN543157*	JN543093*	–	–
*O. stuxbergi*	971	USMMC 00006	Peninsular Malaysia	KX179514*	KX179531*	MN527562	MN527527
	1770	UMIZ 00002	Sumatra, Indonesia	MN528056	MN528065	–	–
	1048	BDMNH	Brunei	KX179515*	KX179532*	MN527563	MN527528
	3251	PNM 041199	Bohol, Philippines	KX179517*	KX179534*	–	–
	3363	PNM 041202	Bohol, Philippines	KX179518*	KX179535*	MN527564	MN527529
	5602	ITBZC IM 00001	Vietnam	KX179519*	KX179536*	–	–
	5605	ITBZC IM 00002	Vietnam	KX179520*	KX179537*	MG958721*	MG971211*
	S891	ASTM-Mo-S891	China (19°56’N)	JN543155*	JN543091*	–	–
*O. typhae*	1064	BNHS 82	West Bengal, India	–	KX179528*	–	–
	1089	BNHS 82-1089	Andaman, India	KX179512*	KX179529*	–	–
	1109	BNHS 21-1109	Andaman, India	KX179513*	KX179530*	–	–
	967	USMMC 00003	Peninsular Malaysia	KX179510*	KX179526*	MN527560	MN527525
	965	USMMC 00005	Peninsular Malaysia	KX179509*	KX179525*	MG958720*	MG971210*
	1007	ZRC.MOL.6396	Singapore	KX179511*	KX179527*	MN527561	MN527526
*Alionchis jailoloensis*	5137	UMIZ 00117	Indonesia, Halmahera	MG953528*	MG953538*	MG953548*	MK122918*
*Marmaronchis vaigiensis*	1183	ZRC.MOL.3007	Singapore	MK122812*	MK122854*	MK122877*	MK122910*
*M. marmoratus*	5409	MNHN-IM-2013-15764	PNG, Madang	MK122838*	MK122859*	MK122893*	MK122915*
*Melayonchis aileenae*	970	USMMC 00018	Peninsular Malaysia	KX240033*	KX240057*	MK122902*	MK125514*
*M. annae*	1010	ZRC.MOL.6502	Singapore	KX240015*	KX240039*	MK122903*	MK122919*
*M. eloisae*	1011	ZRC.MOL.6499	Singapore	KX240026*	KX240050*	MK122904*	MK125515*
*M. siongkiati*	1002	ZRC.MOL.6501	Singapore	KX240020*	KX240044*	MK122905*	MK122920*
*Paromoionchis penangensis*	957	USMMC 00061	Peninsular Malaysia	MH055078*	MH055137*	MH055255*	MH055293*
*P. tumidus*	963	USMMC 00057	Peninsular Malaysia	MH054946*	MH055101*	MH055194*	MH055266*
*Onchidella celtica*	5013	MNHN-IM-2014-6891	France	MG958715*	MG958717*	MK122906*	MK122921*
*O. nigricans*	1524	AM C468921.002	Australia, NSW	MG970878*	MG970944*	MK122908*	MK122923*
*Onchidina australis*	1523	AM C468918.002	Australia, NSW	KX179548*	KX179561*	MG958719*	MG971209*
*Peronia* sp.	706	UF 303653	USA, Hawaii	HQ660038*	HQ659906*	MG958722*	MG971212*
	696	UF 352288	Japan, Okinawa	HQ660043*	HQ659911*	MG958871*	MG958883*
*Peronina tenera*	960	USMMC 00039	Peninsular Malaysia	MG958740*	MG958796*	MG958840*	MG958874*
*P. zulfigari*	924	USMMC 00048	Peninsular Malaysia	MG958760*	MG958816*	MG958853*	MG958876*
*Platevindex luteus*	1001	ZRC.MOL.10179	Singapore	MG958714*	MG958716*	MG958718*	MG958888*
*Wallaconchis ater*	3272	PNM 041222	Philippines, Bohol	MG970809*	MG970910*	MG971132*	MG971185*
*W. sinanui*	2740	UMIZ 00059	Indonesia, Ambon	MG970713*	MG970881*	MG971093*	MG971161*

* Sequences (including all sequences of the outgroups) from our former publications (Dayrat et al. 2011, 2016, 2017, 2018, 2019a, b; Dayrat and Goulding 2017; Goulding et al. 2018a, b, c). Sequences from China were obtained from GenBank (Sun et al. 2014) where they are misidentified as *Onchidium struma*, a *nomen nudum*. Abbreviations: Australian Museum, Sydney (AM); Aquatic Science and Technology Museum of Shanghai Ocean University (ASTM); Brunei Darussalam Museum of Natural History (BDMNH); Bombay Natural History Society, India (BNHS); Institute of Tropical Biology, Zoology Collection, Vietnam Academy of Science and Technology (ITBZC); Muséum national d’Histoire naturelle, Paris, France (MNHN); National Museum of the Philippines, Manila (PNM); University of Florida, Gainesville (UF); Universiti Sains Malaysia Mollusc Collection, Penang, Malaysia (UMIZ); Universiti Sains Malaysia Mollusc Collection, Penang, Malaysia (USMMC); Zoological Reference Collection, Lee Kong Chian Natural History Museum, National University of Singapore (ZRC).

Specimens were individually numbered and photographed in their respective habitat. At each site, we endeavored to sample as much diversity as possible. In addition to numbering individually the specimens that looked different, we also numbered individually specimens that looked similar so that we could test for the presence of cryptic diversity. Importantly, a piece of tissue was cut for all specimens individually numbered (for DNA extraction) and the rest of each specimen was relaxed (using magnesium chloride) and fixed (using 10% formalin or 70% ethanol) for comparative anatomy.

### Specimens

Eighteen specimens were already included in our revision of the genus *Onchidium* and are included in the molecular analyses here to demonstrate the existence of a new species and of a new record for *O. stuxbergi* (Table 1). Their mitochondrial COI and 16S sequences are from our revision of *Onchidium*, but their nuclear ITS2 and 28S sequences are new. All mitochondrial and nuclear sequences for the 10 specimens representing a new species or a new record are new. Overall, mitochondrial COI and 16S sequences are provided for 28 individuals and nuclear 28S and ITS2 sequences are provided for 12 of those 28 individuals (excluding outgroups). All DNA sequences were generated by us except for the mitochondrial sequences of six individuals from China obtained from GenBank (Table 1).

DNA extraction numbers unique to each individual are indicated in phylogenetic analyses as well as lists of material examined and figure captions (numbers are between brackets). Size (length/width) is indicated in millimeters (mm) for each specimen. Many additional specimens were examined in the context of our revision of the family, including all available types (the types of *Onchidium pallidipes* Tapparone-Canefri, 1889 and *Onchidium multinotatum* Plate, 1893, are addressed in detail in the discussion) and hundreds of onchidiids representing all the known genera and nearly all known species. The ten specimens representing a new species and a new record were deposited as vouchers in institutions in the countries of origin: Bombay Natural History Society, Mumbai, (India); Universitas Malikussaleh, North Aceh, Sumatra (Indonesia); Universiti Sains Malaysia, Penang (Malaysia).

### Museum collection abbreviations

**MNHN** Muséum national d’Histoire naturelle, Paris, France;

**NMNH** National Museum of Natural History, Smithsonian Institution, Washington, DC, USA;

**SMNH** Swedish Museum of Natural History, Stockholm, Sweden;

**UMIZ** Universitas Malikussaleh, North Aceh, Sumatra, Indonesia;

**USMMC** Universiti Sains Malaysia, Mollusk Collection, Penang, Malaysia;

**ZMB** Museum für Naturkunde, Berlin, Germany;

**ZMH** Zoologisches Museum, Hamburg, Germany.

### Anatomical preparations and descriptions

Both the external morphology and the internal anatomy were studied. All anatomical observations were made under a dissecting microscope and drawn with a camera lucida. Radulae and male reproductive organs were prepared for scanning electron microscopy (Zeiss SIGMA Field Emission Scanning Electron Microscopy). Radulae were cleaned in 10% NaOH for a week, rinsed in distilled water, briefly cleaned in an ultrasonic water bath (less than a minute), sputter-coated with gold-palladium and examined by SEM. Soft parts (penis, accessory penial gland, etc.) were dehydrated in ethanol and critical point dried before coating.

The detailed anatomy of the type species, *Onchidium typhae*, can be found in our revision of *Onchidium* (Dayrat et al. 2016). To avoid unnecessary repetition, the description of anatomical features that are virtually identical between *Onchidium* species (e.g., position of female opening, position of anus, size of hyponotum relative to total width, nervous system, heart, and stomach) is not repeated here. However, all the characters that are useful for species comparison (e.g., color of live animals, radular formulae, intestinal loops, and reproductive system) are described for the new species. Special attention has been given to illustrating the holotype of the new species and its habitat, including an image of its type locality.

### DNA extraction and PCR amplification

DNA was extracted using a phenol-chloroform extraction protocol with cetyltrimethyl-ammonium bromide (CTAB). The mitochondrial cytochrome *c* oxidase I region (COI) and 16S region were amplified using the following universal primers: LCO1490 (5’-3’) GGT CAA CAA ATC ATA AAG ATA TTG G, and HCO2198 (5’-3’) TAA ACT TCA GGG TGA CCA AAR AAY CA (Folmer et al. 1994), 16Sar-L (5’-3’) CGC CTG TTT ATC AAA AAC AT (Palumbi 1996), and the modified Palumbi primer 16S 972R (5’-3’) CCG GTC TGA ACT CAG ATC ATG T (Dayrat et al. 2011). The nuclear ITS2 region and 28S region were amplified with the following primers: LSU-1 (5’-3’) CTA GCT GCG AGA ATT AAT GTG A, and LSU-3 (5’-3’) ACT TTC CCT CAC GGT ACT TG (Wade and Mordan 2000), 28SC1 (5’-3’) ACC CGC TGA ATT TAA GCA T (Hassouna et al. 1984), and 28SD3 (5’-3’) GAC GAT CGA TTT GCA CGT CA (Vonnemann et al. 2005). The 25 μl PCRs for COI and 16S contained 15.8 μl of water, 2.5 μl of 10X PCR Buffer, 1.5 μl of 25 mM MgCl_2_, 0.5 μl of each 10 μM primer, 2 μl of dNTP Mixture, 0.2 μl (1 unit) of TaKaRa Taq (Code No. R001A), 1 μl of 20 ng/μl template DNA, and 1 μl of 100X BSA (Bovine Serum Albumin). The PCRs for ITS2 used the reagents in the same amounts as COI and 16S, except that water was reduced to 14.8 μl and the amount of 100X BSA was increased to 2 μl. The PCRs for 28S included 14.8 μl of water, 2.5 μl of 10X PCR Buffer, 0.5 μl of each 10 μM primer, 1 μl of dNTP Mixture, 5 μl of Q solution (which includes MgCl_2_) and 0.5 μl of 20 ng/μl template DNA. The thermoprofile used for COI and 16S was: 5 minutes at 94 °C; 30 cycles of 40 seconds at 94 °C, 1 minute at 46 °C, and 1 minute at 72 °C; and a final extension of 10 minutes at 72 °C. The thermoprofile used for ITS2 was: 1 minute at 96 °C; 35 cycles of 30 seconds at 94 °C, 30 seconds at 50 °C, and 1 minute at 72 °C; and a final extension of 10 minutes at 72 °C. The thermoprofile used for 28S was: 4 minutes at 94 °C; 38 cycles of 50 seconds at 94 °C, 1 minute at 52 °C, and 2 minutes 30 seconds at 72 °C; and a final extension of 10 minutes at 72 °C. The PCR products were cleaned with ExoSAP-IT (Affymetrix, Santa Clara, CA, USA) prior to sequencing. Untrimmed sequenced fragments represented approximately 680 bp for COI, 530 bp for 16S, 740 bp for ITS2, and 1000 bp for 28S.

### Phylogenetic analyses

Chromatograms were consulted to resolve rare ambiguous base calls. DNA sequences were aligned using Clustal W in MEGA 7 (Kumar et al. 2016). Nineteen onchidiid species outside *Onchidium* were selected as outgroups from our previous studies (Dayrat et al. 2011, 2016, 2017, 2018, 2019a, b; Dayrat and Goulding 2017; Goulding et al. 2018a, b, c): *Alionchis jailoloensis* Goulding & Dayrat in Goulding et al. 2018a, *Marmaronchis marmoratus* (Lesson, 1831), *Marmaronchis vaigiensis* (Quoy & Gaimard, 1825), *Melayonchis aileenae* Dayrat & Goulding in Dayrat et al. 2017, *Melayonchis annae* Dayrat in Dayrat et al. 2017, *Melayonchis eloisae* Dayrat in Dayrat et al. 2017, *Melayonchis siongkiati* Dayrat & Goulding in Dayrat et al. 2017, *Onchidella celtica* (Cuvier in Audouin and Milne-Edwards 1832), *Onchidella nigricans* (Quoy & Gaimard, 1832), *Onchidina australis* (Semper, 1880), *Paromoionchis daemelii* (Semper, 1880), *Paromoionchis tumidus* (Semper, 1880), *Peronia* sp. (Hawaii), *Peronia* sp. (Okinawa), *Peronina tenera* (Stoliczka, 1869), *Peronina zulfigari* Goulding & Dayrat in Goulding et al. 2018c, *Platevindex luteus* (Semper, 1880), *Wallaconchis ater* (Lesson, 1831), and *Wallaconchis sinanui* Goulding & Dayrat in Goulding et al. 2018b. All new DNA sequences were deposited in GenBank and vouchers deposited in museum collections (Table 1). The ends of each alignment were trimmed. Alignments of mitochondrial (COI and 16S) sequences and nuclear (ITS2 and 28S) sequences were concatenated separately in order to test whether these two data sets support the same relationships. The concatenated mitochondrial alignment included 986 nucleotide positions: 582 (COI) and 404 (16S). The concatenated ITS2 and 28S alignment included 1467 nucleotide positions: 472 (ITS2) and 995 (28S).

Two independent sets of phylogenetic analyses were performed: 1) Maximum Likelihood and Bayesian analyses with concatenated mitochondrial COI and 16S sequences; 2) Maximum Parsimony analyses with concatenated nuclear ITS2 and 28S sequences. Maximum Parsimony analyses were conducted in PAUP v 4.0 (Swofford 2002) with gaps coded as a fifth character state, and 100 bootstrap replicates conducted using a full heuristic search. Prior to Maximum Likelihood and Bayesian phylogenetic analyses, the best-fitting evolutionary model was selected for each locus separately using the Model Selection option from Topali v2.5 (Milne et al. 2004): a GTR + G model was independently selected for COI and 16S. Maximum Likelihood analyses were performed using PhyML (Guindon and Gascuel 2003) as implemented in Topali. Node support was evaluated using bootstrapping with 100 replicates. Bayesian analyses were performed using MrBayes v3.1.2 (Ronquist and Huelsenbeck 2003) as implemented in Topali, with five simultaneous runs of 1.5×10^6^ generations each, sample frequency of 100, and burn in of 25% (and posterior probabilities were also calculated). Topali did not detect any issue with respect to convergence. All analyses were run several times and yielded the same result.

In addition, another set of analyses was performed with only COI sequences. Genetic distances between COI sequences were calculated in MEGA 7 as uncorrected p-distances. COI sequences were also translated into amino acid sequences in MEGA using the invertebrate mitochondrial genetic code to check for the presence of stop codons (no stop codon was found).

## Results

### Molecular phylogenetic analyses (Figs 1, 2)

DNA sequences were used to test species limits within *Onchidium*. The monophyly of *Onchidium* is recovered in all analyses. In the analyses based on mitochondrial COI and 16S concatenated sequences, four least-inclusive units are reciprocally monophyletic: *O. reevesii*, *O. typhae*, *O. stuxbergi* and the new species, *O. melakense*. The monophyly of each species is strongly supported by a bootstrap support of 98 or higher and a posterior probability of 1. Analyses with nuclear 28S and ITS2 concatenated sequences yielded similar results: *O. typhae*, *O. stuxbergi*, and the new species *O. melakense* are strongly supported with bootstrap values of 100. *Onchidium reevesii* could not be included in the nuclear analyses because ITS2 sequences for the specimens from China are not available in GenBank (Table 1).

**Figure 1.**
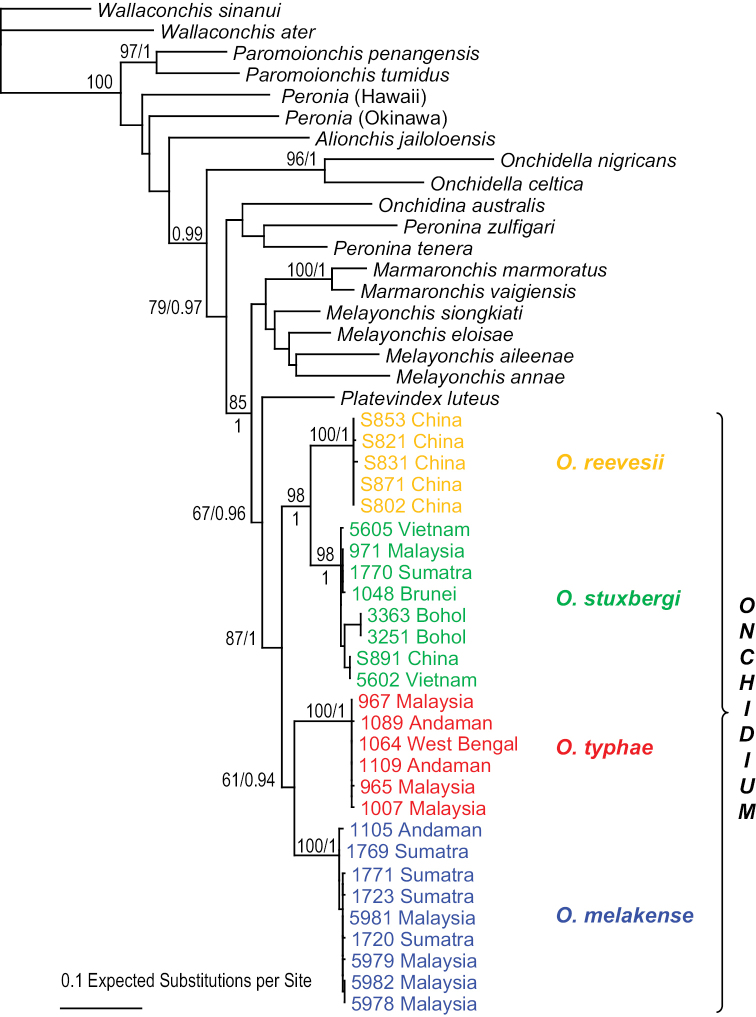
Phylogenetic tree showing the relationships between *Onchidium* individuals based on mitochondrial COI and 16S DNA sequences. Numbers by the nodes are the bootstrap values (Maximum Likelihood analysis) and the posterior probabilities (Bayesian analysis); only significant numbers (> 65% and > 0.9) are indicated. All other sequences serve as outgroups. Information on specimens can be found in the lists of material examined and Table 1. The colors used for each *Onchidium* species are the same as those used in Figs 2–4.

**Figure 2.**
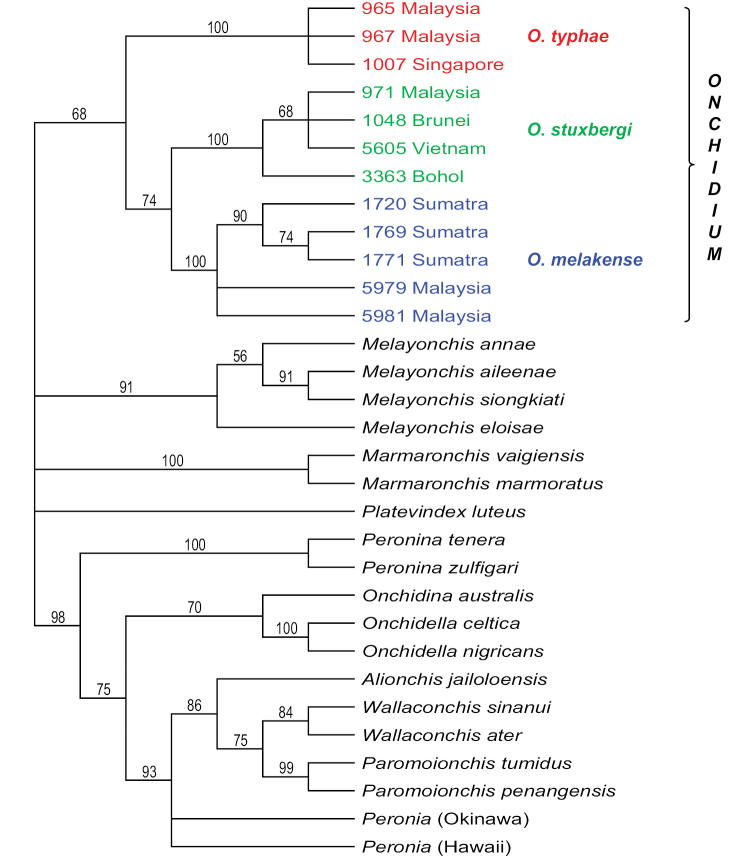
Consensus tree showing relationships between *Onchidium* individuals based on concatenated nuclear ITS2 and 28S sequences. Numbers by the nodes are the bootstrap values (Maximum Parsimony analysis); only significant numbers (> 50%) are indicated. All other sequences serve as outgroups. Information on specimens can be found in the lists of material examined and Table 1. The colors used for each *Onchidium* species are the same as those used in Figs 1, 3, and 4.

### Pairwise genetic divergences (Fig. 3)

Pairwise genetic distances (between COI sequences) support the existence of four species of *Onchidium* as least-inclusive molecular units (Table 2). The intra-specific genetic distances are all below 3.2% (within *O. stuxbergi*). The inter-specific distances vary from 8.6% (between *O. reevesii* and *O. stuxbergi*) to 14.3% (between *O. reevesii* and *O. typhae*). So, overall, the distance gap between the four *Onchidium* species is between 3.2% and 8.6%.

**Figure 3.**
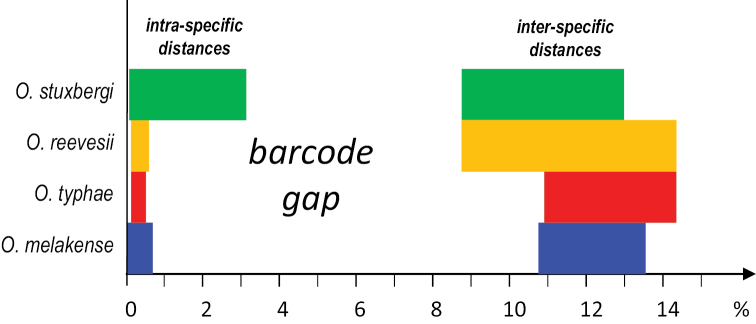
Diagram to help visualize pairwise genetic distances between COI sequences within and between *Onchidium* species (Table 2). Ranges of minimum to maximum distances are indicated (in percentages). For instance, the intra-specific divergences within *O. typhae* are between 0.1 and 0.4%, while the inter-specific divergences between *O. typhae* and the three other species are between 10.9 and 14.3%. Overall, the distance gap between all four *Onchidium* species is between 3.2 and 8.6%. The colors used for each *Onchidium* species are the same as those used in Figs 1, 2, and 4.

**Table 2.** Pairwise genetic distances between mitochondrial COI sequences in *Onchidium*. Ranges of minimum to maximum distances are indicated (in percentage). For instance, the intra-specific divergences within *O. typhae* are between 0.1 and 0.4%, while the inter-specific divergences between *O. typhae* and *O. stuxbergi* are between 11.4 and 12.9%.

**Species**	***O. typhae***	***O. stuxbergi***	***O. melakense***	***O. reevesii***
*O. typhae*	0.1–0.4	–	–	–
*O. stuxbergi*	11.4–12.9	0.0–3.2	–	–
*O. melakense*	10.9–11.9	10.7–13.0	0.0–0.7	–
*O. reevesii*	13.4–14.3	8.6–10.1	12.6–13.5	0.1–0.5

### Comparative anatomy

Due to its distinctive external color, the new species was immediately recognized in the field as new to science. It also differs in internal anatomy from the three other known species. In particular, the penial sheath in the male copulatory apparatus is short and straight while coiled in the three other species (Table 3).

**Table 3.** Morphological differences among *Onchidium* species. All traits are subject to individual variation. Information regarding *O. stuxbergi* and *O. typhae* is from Dayrat et al. (2016). Information regarding *O. reevesii* is from Dayrat et al. (2016) for the holotype, and from Wang et al. (2018) for non-type material. For the type of intestinal loops, the orientation of the transitional loop (TL) is provided. For the radular formulae, the range of number of rows (e.g., 60 to 80 rows in *O. melakense*) and the range of number of lateral teeth per half row (e.g., 70 to 110 in *O. melakense*) are provided. The number of radular rows was not described in *O. reevesii* by Wang et al. (2018).

**Species**	***O. melakense***	***O. reevesii***	***O. stuxbergi***	***O. typhae***
Size	Up to 45 mm	Up to 67 mm	Up to 55 mm	Up to 65 mm
Dorsal color	Light brown	Brown	Brown, occasionally black	Brownish
Foot color	Pale yellow-beige	Whitish or light yellow	Bright orange	Grey to yellow, sometimes greenish
Hyponotum color	White	Light grey or beige-white	Greyish to yellowish, sometimes greenish	Grey to yellow, sometimes greenish
Black dots on hyponotum	Absent	Present	Present	Absent
Type of intestinal loops	III, TL from 1 to 5 o’clock	III, TL at 2 o’clock	III, TL from 1 to 8 o’clock	II, TL from 8 to 9 o’clock
Radular formulae	60/80, 70/110	62/110 (lateral teeth only)	50/70, 68/80	53/65, 65/80
Penial gland spine length	Up to 1.1 mm	No data available	Up to 2 mm	Up to 1.2 mm
Penial sheath	Short and straight	Long and heavily coiled in spirals	Long and heavily coiled in spirals	Long and heavily coiled in spirals
Insertion of retractor muscle in visceral cavity	Middle	Posterior third	Posterior third	Near the heart (India) & posterior half (everywhere else)
Anterior retractor muscle	Present (occasionally absent)	Absent	Present (possibly occasionally absent)	Absent

### Systematics and anatomical descriptions

#### Family Onchidiidae Rafinesque, 1815

##### Genus Onchidium

Taxon classificationAnimaliaSystellommatophoraOnchidiidae

Buchannan, 1800

F2E03CFF-409E-5BD6-A067-DDFFCA535463

Onchidium Buchannan, 1800: 132.
Labbella Starobogatov, 1970: 45; Starobogatov 1976: 211. Replacement name for Elophilus Labbé, 1935, preoccupied by Elophilus Meigen, 1803 [Diptera].

###### Type species.

*Onchidium typhae* Buchannan, 1800, by monotypy.

###### Gender.

Neuter, gender of the final component of *Onchidium*, a name formed from the masculine Greek word ὁ ὂγκος (mass, tumor) and the neuter Latin suffix -*ium* (ICZN 1999: Article 30.1.1).

###### Diagnosis.

Body not flattened. No dorsal gills. Dorsal eyes present on notum. Large, conical, pointed papillae present on notum. Retractable, central papilla (with three or four dorsal eyes) present but not significantly larger than surrounding papillae. Eyes at tip of extremely long ocular tentacles. Male opening below right ocular tentacle and slightly to its left. Transversal protuberance on oral lobes present. Foot wide. Pneumostome medial, on average in middle between foot margin and notum margin. Intestinal loops of types II and III. Rectal gland present. Accessory penial gland present with a hollow spine but no muscular sac. Penis with hooks.

###### Distinctive features.

In the field, *Onchidium* slugs differ from all other onchidiids by the presence of large, conical, pointed papillae on the dorsal notum. However, papillae can only be observed when animals remain undisturbed. In disturbed (and preserved) animals, papillae remain pointed but become minute. However, the best feature to identify *Onchidium* slugs in the field is the presence of very long and thin ocular tentacles (up to 20 mm). Papillae can definitely be confused between genera but *Onchidium* slugs are (almost) the only ones with such long eye tentacles. Very long ocular tentacles are also present in *Alionchis jailoloensis* but they are much thicker (in diameter) than those of *Onchidium*. Also, *Alionchis jailoloensis* is so far only known from Halmahera (where *Onchidium* is not found) and lacks the large, conical, pointed papillae that are typical of *Onchidium*. Finally, in *Alionchis*, the pneumostome is always located exactly at the margin of the notum. Therefore, *Onchidium* slugs cannot be confused with *Alionchis* slugs.

###### Distribution.

**Figure 4.**
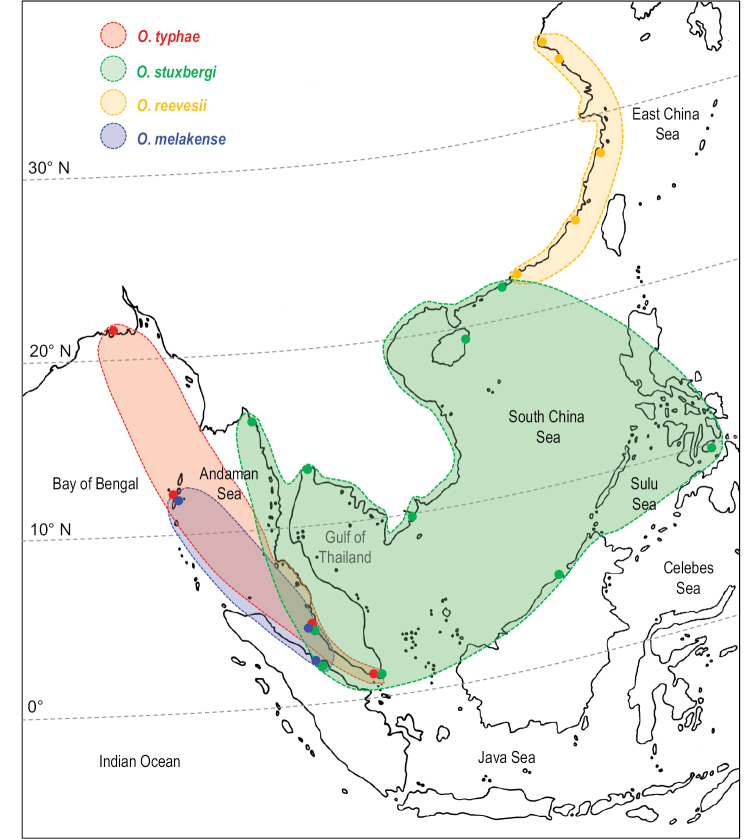
Geographic distribution of the four known *Onchidium* species. Dots correspond to known records. The colors used for each *Onchidium* species are the same as those used in Figs 1–3.

From northeastern India (West Bengal) to the Philippines, including the Strait of Malacca, Singapore, Thailand, Vietnam, eastern Borneo, and China (Fig. 4).

###### Remarks.

The diagnosis and the distinctive features provided above are slightly updated from Dayrat et al. (2016). The synonymy of *Labbella* (replacement name for *Elophilus*) with *Onchidium* was already discussed by Dayrat et al. (2016). In brief, *Labbella ajuthiae* (Labbé, 1935), the type species of *Labbella*, is a junior synonym of *Onchidium stuxbergi* (Westerlund, 1883). Therefore, both *Labbella* and *Onchidium* apply to the same clade. We remark a detail concerning the nomenclatural status of *Elophilus* Meigen, 1803. Under plenary powers of the Commission (ICZN 1993: 256), the generic name *Elophilus* Meigen, 1803 was “suppressed for the purposes of the Principle of Priority but not for those of the Principle of Homonymy.” So, even though *Elophilus* Meigen, 1803, was placed on the Official Index of Rejected and Invalid Generic Names in Zoology, *Elophilus* Labbé, 1935 remains a junior homonym of *Elophilus* Meigen, 1803, hence the necessity of the replacement name *Labbella*. Also, note that the publication date for *Labbella* by Starobogatov is 1970 instead of 1976 (Dayrat 2009; Dayrat et al. 2016).

##### Onchidium melakense

Taxon classificationAnimaliaSystellommatophoraOnchidiidae

Dayrat & Goulding sp. nov.

9E20AE87-9F32-56C4-962F-47F580768879

http://zoobank.org/D7C0CD7A-C888-407B-9E36-C1544835B355

Figs 5 , 6 , 7 , 8 , 9 , 10 , 11 , 13G, H

###### Type material.

***Holotype*.** Malaysia • holotype, designated here, 45/25 mm [5979 H]; Peninsular Malaysia, Kuala Sepatang; 04°50.605'N, 100°38.133'E; 28 Jul 2016; B Dayrat and field party leg.; st 258, old forest with tall *Rhizophora* trees, high in the tidal zone (ferns), in educational mangrove preserve; USMMC 00075.

###### Additional material examined.

India – **Andaman Islands** • 1 specimen 25/15 mm [1105]; Middle Andaman, Rangat, Shyamkund; 12°28.953'N, 92°50.638'E; 11 Jan 2011; B Dayrat and field party leg.; st 57, by a large river, deep mangrove with tall trees, small creeks, and many muddy logs; BNHS 94. Malaysia – **Peninsular Malaysia** • 3 specimens 30/20 mm [5978], 35/18 mm [5981], and 35/30 mm [5982]; same collection data as for the holotype; USMMC 00076. Indonesia – **Sumatra** • 4 specimens 27/20 mm [1723], 25/22 mm [1720], 35/20 mm [1769], and 40/20 mm [1771]; Pulau Sinaboi; 02°18.145'N, 100°59.309'E; 8 Oct 2012; M Khalil and field party leg.; st 73, mangrove forest with medium *Rhizophora* and *Avicennia* trees, logs, hard mud; UMIZ 00001.

###### Distribution

(Fig. 4). Western Peninsular Malaysia (type locality), eastern Sumatra (Indonesia), and Andaman Islands (India).

###### Etymology.

*Onchidium melakense* is named after the Strait of Malacca or ‘Selat Melaka’ in Malay: *melakense* is a Latinized adjective that agrees in gender (neuter) with the generic name (ICZN 1999: Art. 31.2). The mangrove gastropod diversity of the Strait of Malacca is extraordinarily rich. For instance, three of the four known *Onchidium* species are sympatric there: *O. typhae*, *O. stuxbergi*, and the new species *O. melakense*. The fourth species, *O. reevesii*, is restricted to the Chinese coast (Fig. 4).

###### Habitat

**Figure 5.**
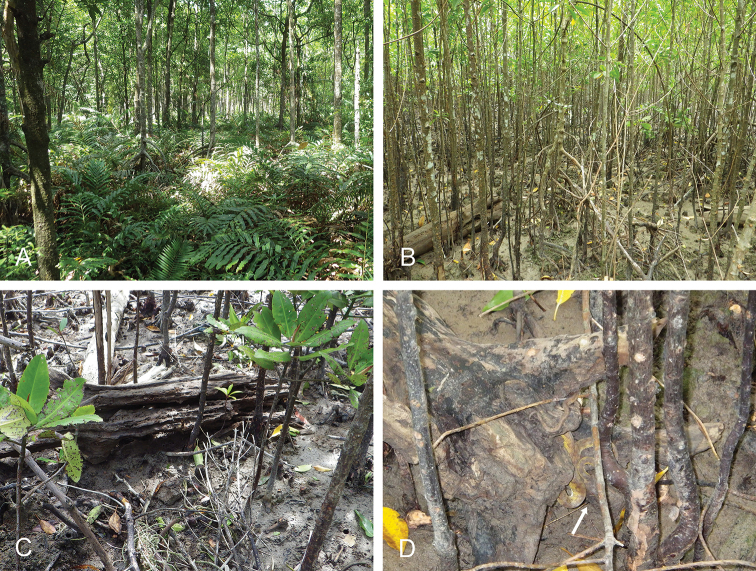
Habitats, *Onchidium melakense***A** type locality, Peninsular Malaysia, Kuala Sepatang, old forest with tall *Rhizophora* trees, high in the tidal zone (ferns), in educational mangrove preserve (st 258) **B** Sumatra, Pulau Sinaboi, mangrove forest with medium *Rhizophora* and *Avicennia* trees, dead logs, hard mud (st 73) **C** old log with crevices which *O. melakense* typically favors (st 73) **D** mangrove pit viper (arrow) resting by a log (st 73).

(Fig. 5). *Onchidium melakense* was found under a log (type locality, Peninsular Malaysia), inside crevices of a muddy log (Sumatra) and on the cemented wall of a bridge over a mangrove creek (Andaman Islands). Most individuals were hidden and could not have been found if logs had not been turned over and thoroughly searched inside. This search, however, should be done with caution because pit vipers often like to rest near logs in mangroves (Fig. 5D). *Onchidium melakense* does not seem to particularly favor the habitat where *O. typhae* and *O. stuxbergi* are most commonly found, i.e., the surface of muddy trunks, logs, and *Thalassina* lobster mounds. Even though *O. typhae* and *O. stuxbergi* can be found at the same sites as *O. melakense* (they are found in the Matang mangrove, where the type locality of *O. melakense* is located), they do not share exactly the same micro-habitats. Clearly, all these species hide in crevices at high tide but, unlike *O. typhae* and *O. stuxbergi*, *O. melakense* appears to remain hidden at low tide as well.

###### Abundance.

*Onchidium melakense* is a rare species. In total, we found only nine individuals: four individuals at the type locality in Peninsular Malaysia, four individuals at one site in eastern Sumatra, and a single individual in the Andaman Islands.

###### Color and external morphology of live animals

**Figure 6.**
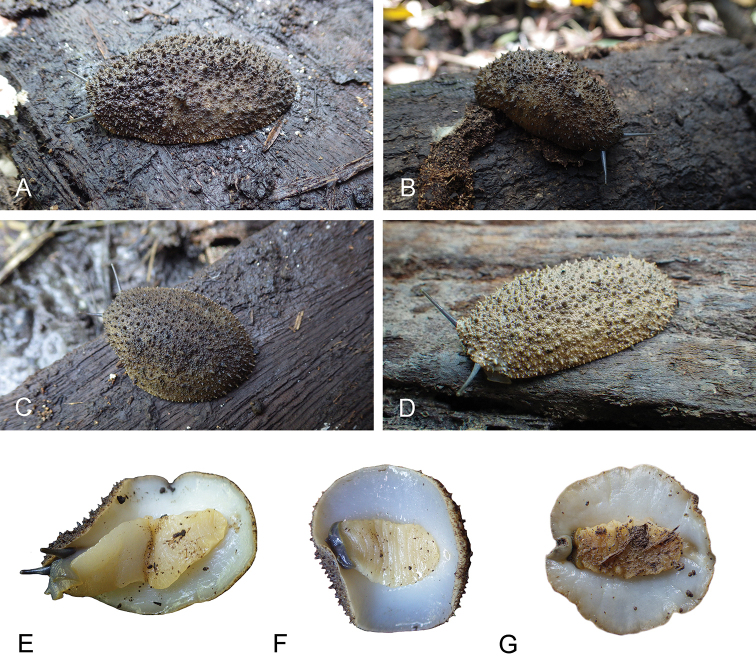
Live animals, *Onchidium melakense***A** dorsal view, holotype, 45 mm long [5979], Peninsular Malaysia (USMMC 00075) **B** dorsal view, 30 mm long [5978], Peninsular Malaysia (USMMC 00076) **C** dorsal view, 35 mm long [5981], Peninsular Malaysia (USMMC 00076) **D** dorsal view, 35 mm long [1769], Sumatra (UMIZ 00001) **E** ventral view, same as A **F** ventral view, same as C **G** dorsal view, 40 mm long [1771], Sumatra (UMIZ 00001).

**Figure 7.**
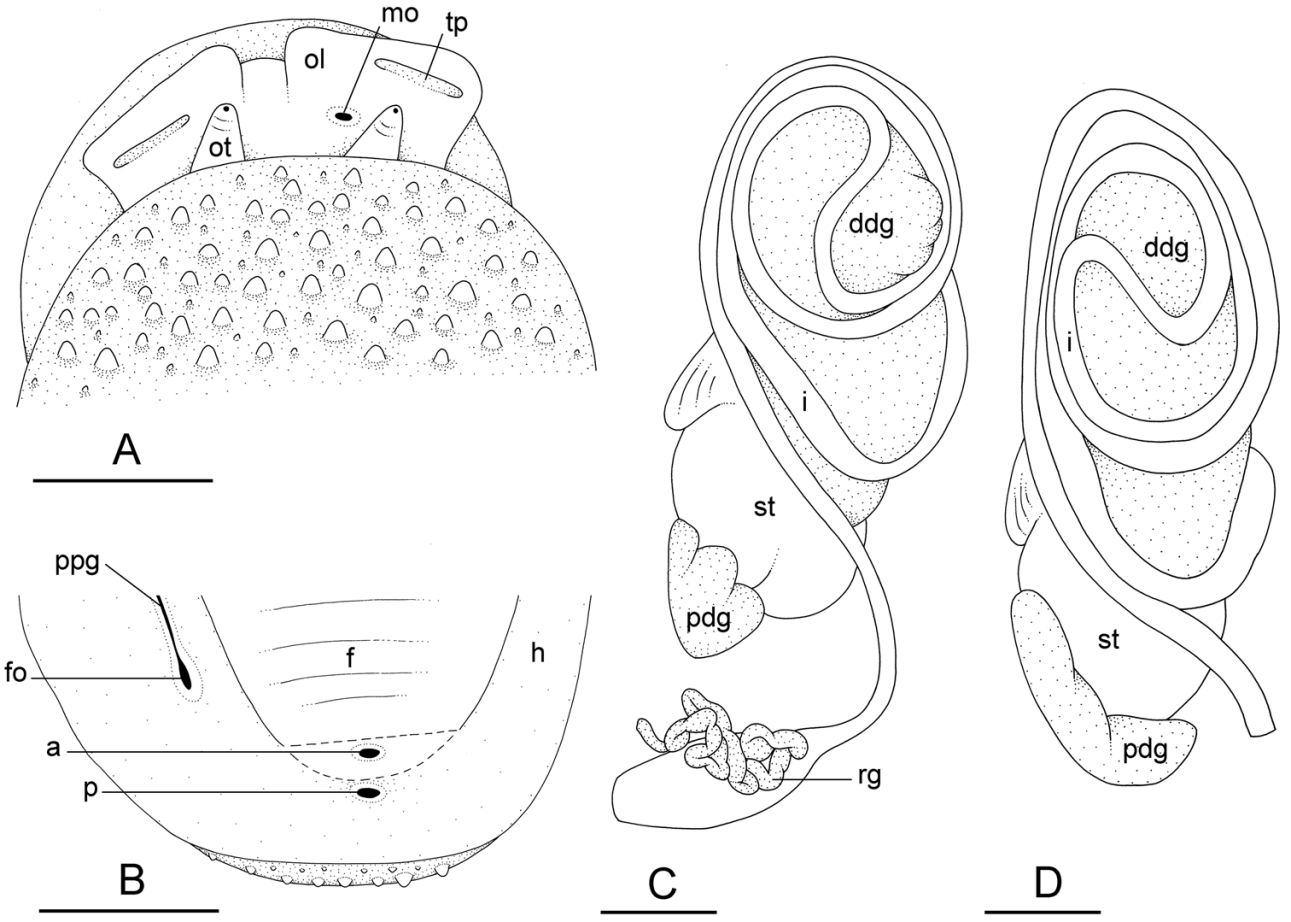
External morphology and digestive system, *Onchidium melakense*, Peninsular Malaysia **A–C** holotype [5979] (USMMC 00075) **D** [5978] (USMMC 00076) **A** dorsal, anterior view **B** posterior, ventral view (dotted lines indicate where the foot was cut to show the anus) **C** digestive system, dorsal view **D** digestive system, dorsal view. Abbreviations: a anus, ddg dorsal lobe of digestive gland, f foot (pedal sole), fo female opening, h hyponotum, i intestine, mo male opening, ol oral lobe, ot ocular tentacle, p pneumostome, pdg posterior lobe of the digestive gland, ppg peripodial groove, rg rectal gland, st stomach, tp transversal protuberance (on oral lobe). Scale bars: 5 mm (**A–C**), 3 mm (**D**).

(Figs 6, 7). Live animals are not covered with mud and the color of their dorsum can normally be seen. The dorsum is homogenously light brown. The hyponotum is distinctly white. The foot is pale yellow-beige. The ocular tentacles are dark grey and are extremely long (up to 2 cm) when animals are undisturbed. The head is grey. Large, conical, pointed papillae (which are typical of *Onchidium* species) are present but can only be seen when the animal remains undisturbed for a long time. Some of these papillae bear dorsal eyes. When animals are disturbed, papillae immediately retract and become minute (although they remain pointed). A central papilla (with three or four dorsal eyes), fully retractable within the dorsal notum, is also present but is not particularly more prominent than surrounding papillae. Crawling individuals are up to 45 mm long. Preserved specimens no longer display the distinct color seen in live animals: the dorsal notum remains light brown and the hyponotum remains white, but the foot of preserved animals is whitish.

###### Digestive system

(Figs 7, 9). Examples of radular formulae are presented in Table 4. The median cusp of the rachidian tooth is always present; its lateral cusps (on its lateral sides) can be conspicuous. The intestine is of type III, with a transitional loop oriented to the right, approximately from 1 to 5 o’clock (for a comparison of intestinal types between *Onchidium* species, see the Discussion).

**Figure 8.**
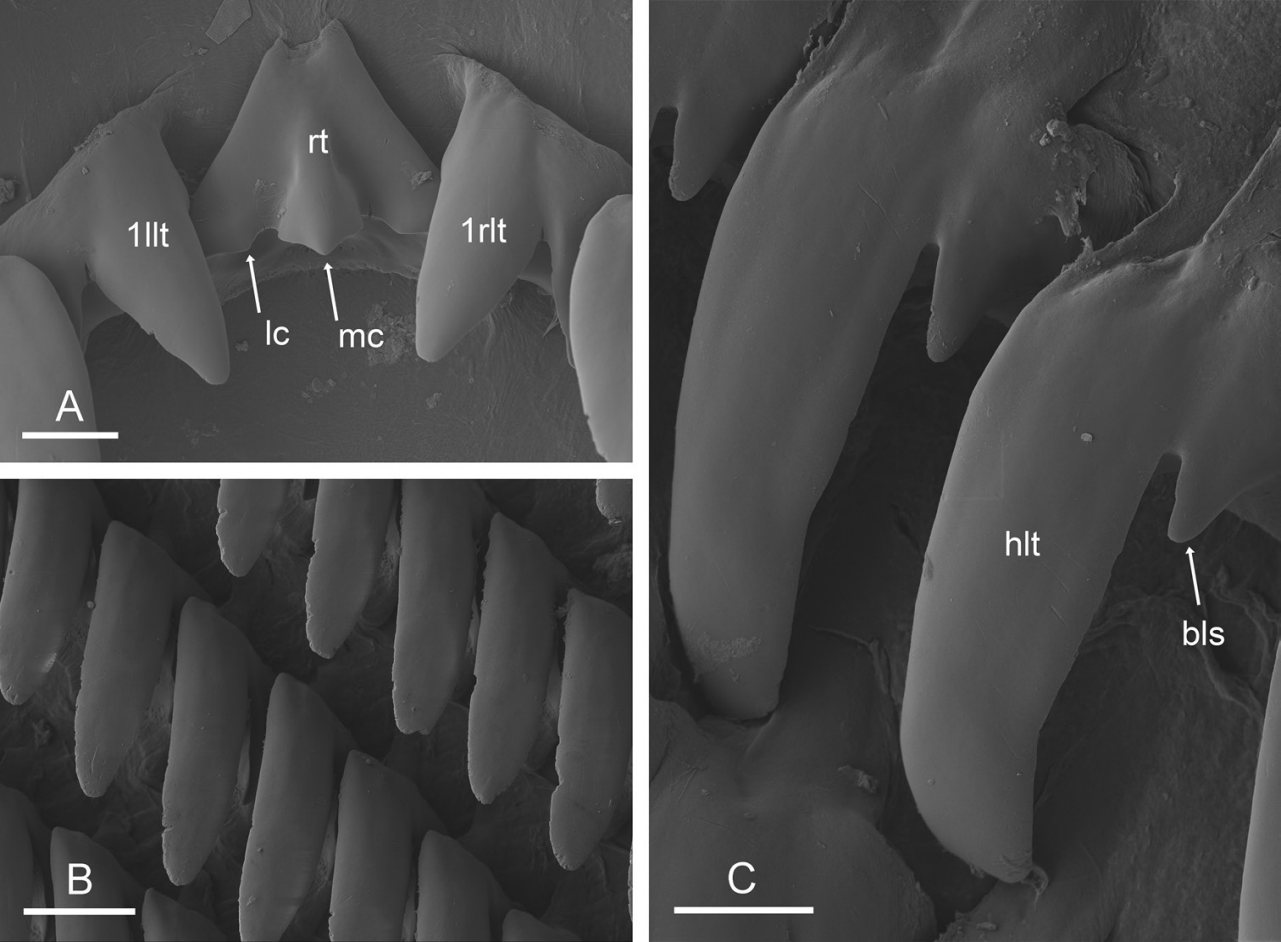
Radula, *Onchidium melakense*, Peninsular Malaysia, holotype [5979] (USMMC 00075) **A** rachidian and innermost lateral teeth **B** right lateral teeth **C** right lateral teeth. Abbreviations: 1llt first left lateral tooth, 1rlt first right lateral tooth, bls basal lateral spine, hlt hook of lateral tooth, lc lateral cusp of rachidian tooth, mc median cusp of rachidian tooth, rt rachidian tooth. Scale bars: 10 μm (**A**,) 20 μm (**B, C**).

**Figure 9.**
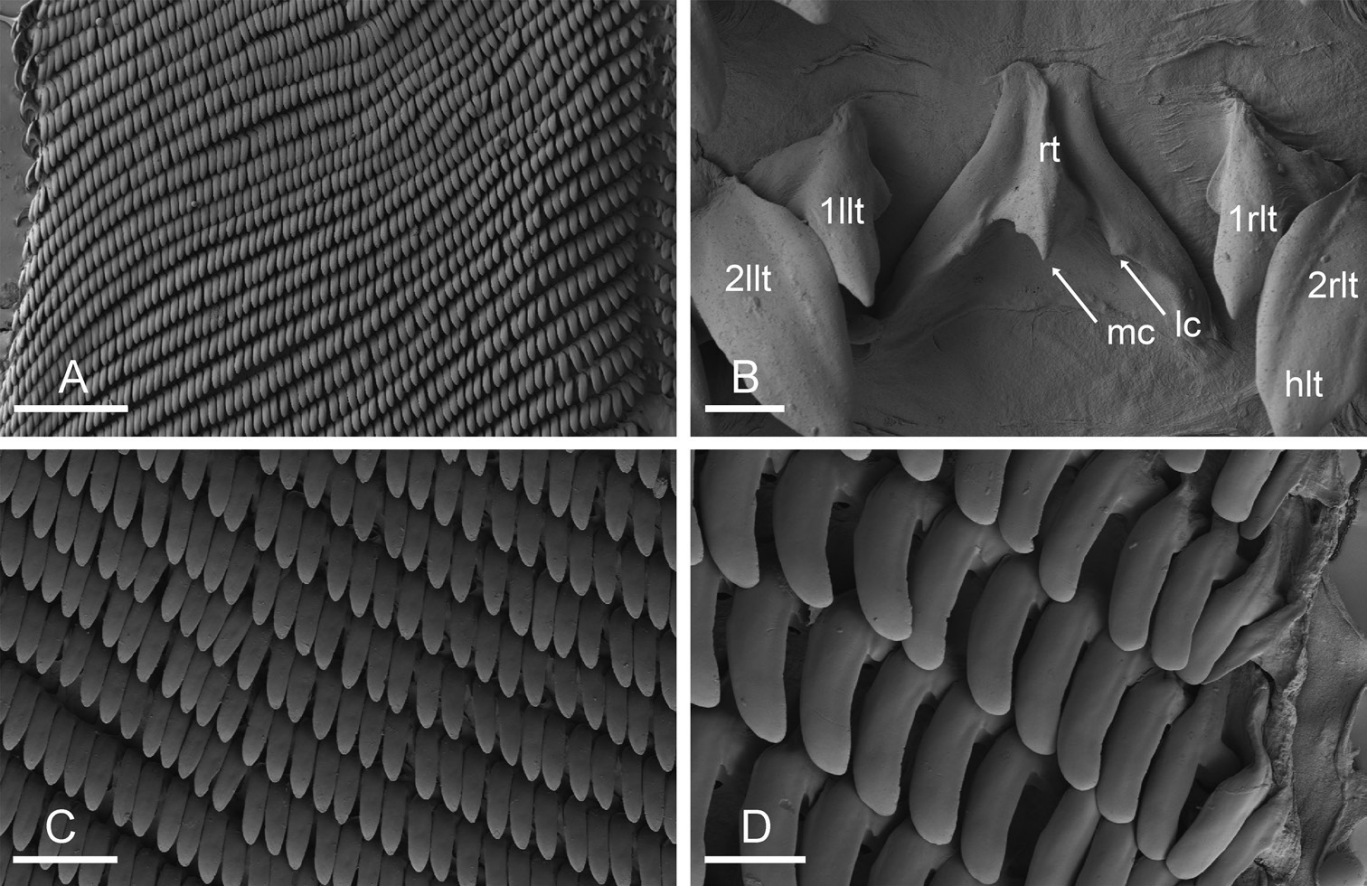
Radula, *Onchidium melakense*, Sumatra (UMIZ 00001) **A** left, half rows, [1720] **B** rachidian and innermost lateral teeth, [1720] **C** lateral teeth, [1723] **D** outermost, lateral teeth, [1723]. Abbreviations: 1llt first left lateral tooth, 1rlt first right lateral tooth, 2llt second left lateral tooth, 2rlt second right lateral tooth, hlt hook of lateral tooth, lc lateral cusp of rachidian tooth, mc median cusp of rachidian tooth, rt rachidian tooth. Scale bars: 200 μm (**A**), 10 μm (**B**), 60 μm (**C**), 20 μm (**D**).

**Table 4.** Radular formulae for *Onchidium melakense*. Each formula follows the same format: number of rows × number of lateral teeth per left half row – 1 (rachidian tooth) – number of lateral teeth per right half row. Each DNA extraction number corresponds to one individual. The letter H next to an extraction number indicates the holotype.

**DNA extraction number**	**Voucher**	**Radular formula**	**Specimen length (mm)**
5979 H	USMMC 00075	80 × 110-1-110	45
5982	USMMC 00076	80 × 90-1-90	35
1723	UMIZ 00001	75 × 90-1-90	27
5981	USMMC 00076	65 × 70-1-70	35
1720	UMIZ 00001	60 × 85-1-85	25

###### Reproductive system

**Figure 10.**
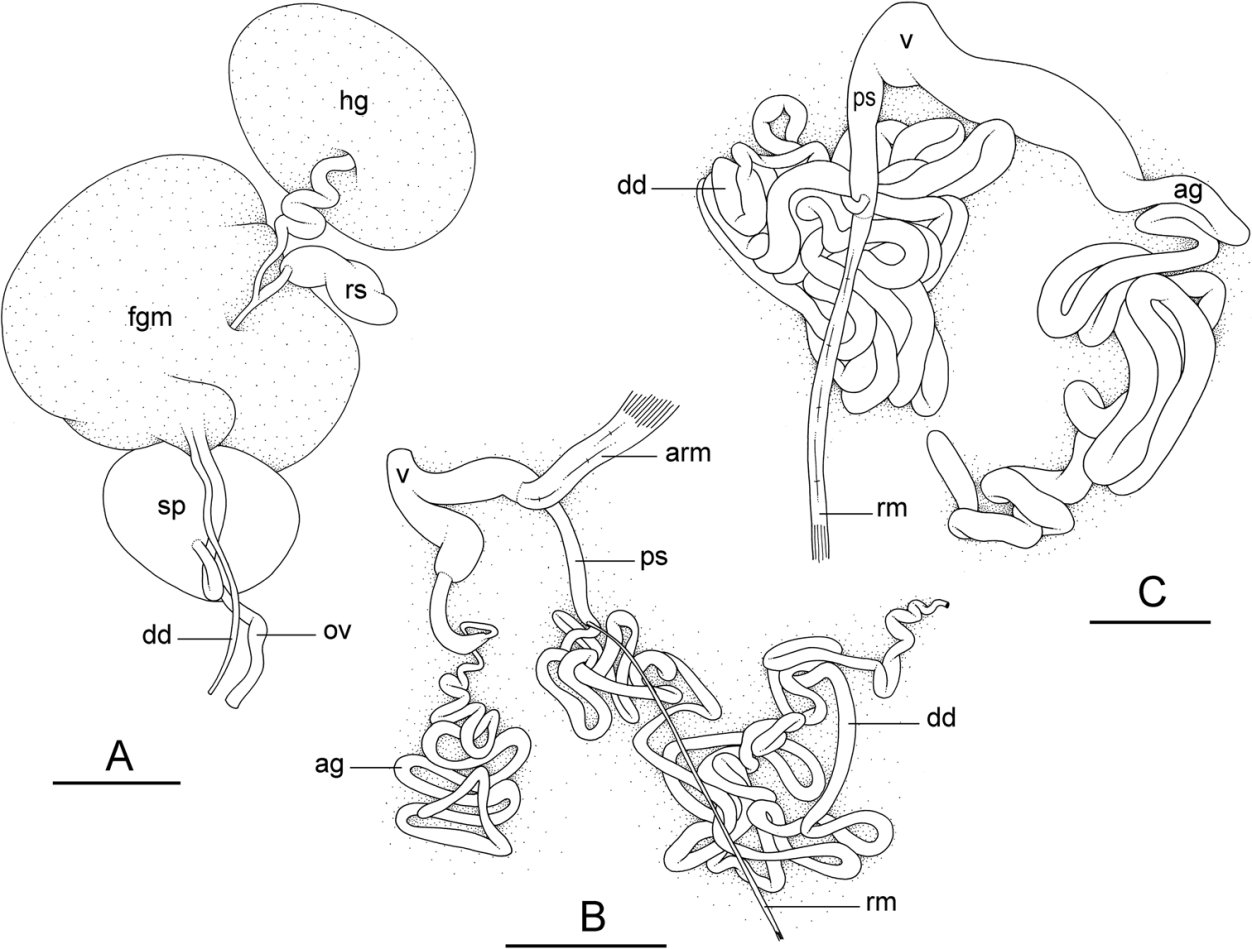
Reproductive system, *Onchidium melakense***A, B** Peninsular Malaysia, holotype [5979] (USMMC 00075) **C** Sumatra, [1723] (UMIZ 00001) **A** posterior, hermaphroditic, reproductive parts **B** anterior, male, copulatory parts **C** anterior, male, copulatory parts. Abbreviations: ag accessory penial gland, arm anterior penial retractor muscle, dd deferent duct, fgm female gland mass, hg hermaphroditic gland, ov oviduct, ps penial sheath, rm penial retractor muscle, rs receptaculum seminis, sp spermatheca, v vestibule. Scale bars: 4 mm (**A**), 5 mm (**B**), 2 mm (**C**).

(Fig. 10A). The receptaculum seminis (caecum) is bent, ovate and elongated. The spermatheca is spherical-ovate and connects to the oviduct through a short duct with one loop. The oviduct and the deferent duct are narrow and straight. A vaginal gland is absent.

###### Copulatory apparatus

**Figure 11.**
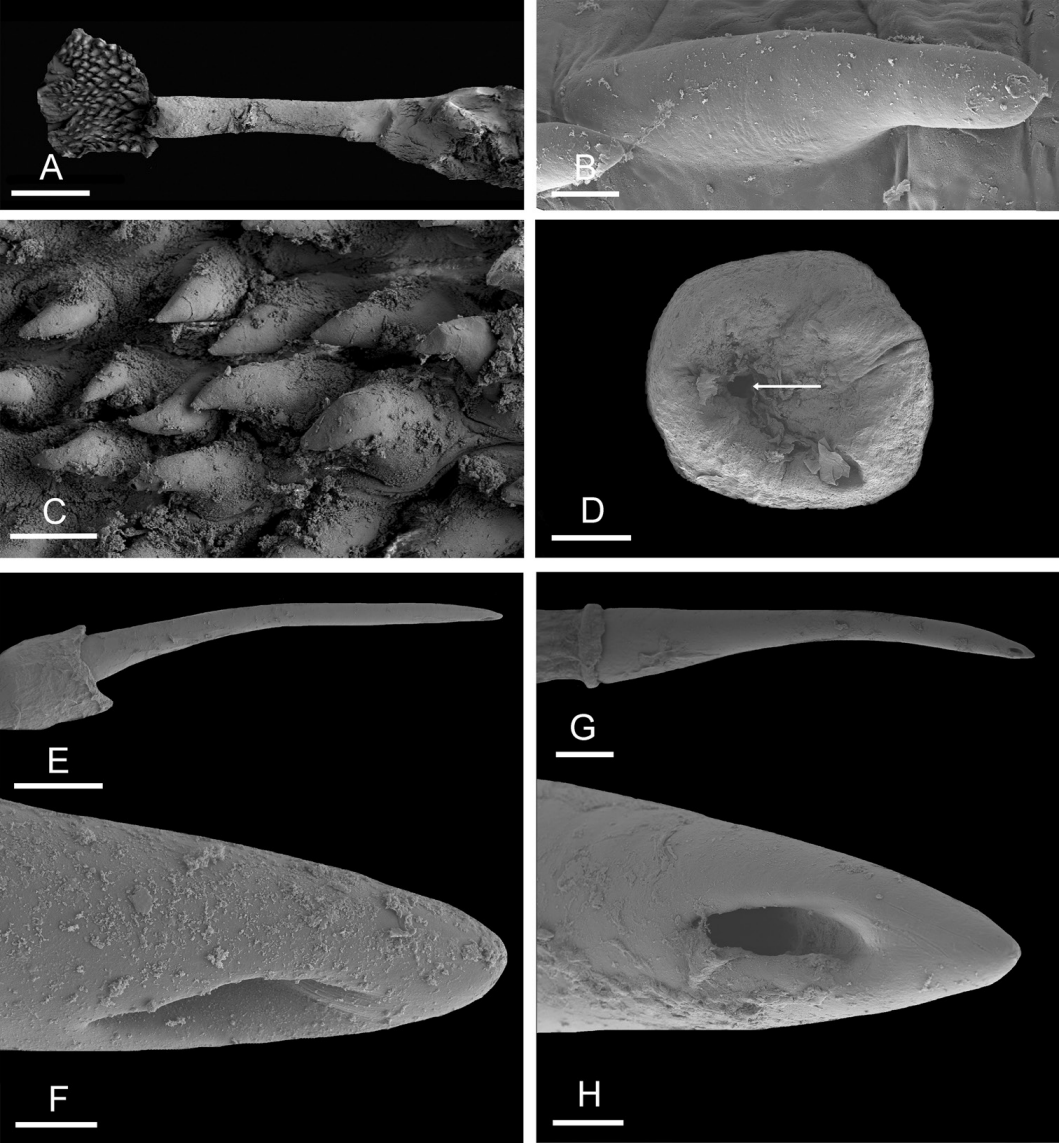
Male, anterior, copulatory parts, *Onchidium melakense***A, C** Sumatra, [1723] (UMIZ 00001) **B, E, F** Peninsular Malaysia, holotype [5979] (USMMC 00075) **D, G, H** Peninsular Malaysia, holotype [5982] (USMMC 00076) **A** stalk and penial hooks **B** penial hook **C** penial hooks **D** flat disc at distal end of flagellum of penial accessory gland (the arrow indicates the hole through which the hollow spine protrudes) **E** hollow spine **F** hollow spine tip **G** hollow spine **H** hollow spine tip. Scale bars: 300 μm (**A**), 10 μm (**B, C, F**), 100 μm (**D, G, H**), 200 μm (**E**).

(Figs 10, 11). The male anterior organs consist of the penial complex (penial papilla, penial sheath, deferent duct, and retractor muscle) and the accessory penial gland (flagellum and hollow spine). The penial complex and the accessory penial gland share the same vestibule and male opening. The flagellum of the penial gland is coiled. Distally, it ends in a hard, hollow spine. The hollow spine is narrow, elongated, and slightly curved. Its length varies from 0.8 to 1.1 mm. Its diameter is approximately 50 μm for most of its length (but approximately 140 μm at its conical base). The hollow spine does not open directly into the proximal region of the vestibule. There is a transversal, flat disc at the distal end of the flagellum (approximately 0.4 mm in diameter) through which the hollow spine must protrude in order to be outside and shared with the partner (Fig. 11D).

The penial sheath is short (less than 5 mm) and straight, not coiled in spirals. The (posterior) retractor muscle is longer than the penial sheath and inserts at about the middle of the visceral cavity floor. An additional, anterior retractor muscle is present (and occasionally absent) in the distal part of the penial sheath. The deferent duct is highly convoluted with many loops. The penis is made of two distinct parts. The proximal part is a hollow, solid, flexible stalk with no hooks; its length varies from 1.2 to 1.8 mm and its diameter from 100 μm to 200 μm. The distal part is short (up to approximately 0.8 mm long), soft, and covered with penial hooks internally. Penial hooks are inside the tube-like penis when the penis is retracted inside the penial sheath. During copulation, the penis is everted like a glove and the hooks are then on the outside. Penial hooks are conical, curved, pointed, and up to 60 μm long.

###### Diagnostic features.

Externally, *Onchidium melakense* differs from all other *Onchidium* species by its color. *Onchidium melakense* is the only known species with a light brown dorsal notum, a pale yellow-beige foot, and a white hyponotum (see Table 3 and the Identification key). Internally, *O. melakense* is the only known species with a short and straight penial sheath, while in other species the penial sheath is long and coiled in spirals (Table 3). Other traits are helpful as well but may not be as diagnostic as the penial sheath. For instance, intestinal loops help distinguish *O. melakense* from *O. typhae* but not from *O. stuxbergi* (Table 3).

###### Remarks.

A new species name is needed because no existing name applies to the species described here, based on the examination of all the type specimens available in the Onchidiidae, a careful study of all the original descriptions, and our ongoing taxonomic revision of every genus of the family (Dayrat et al. 2016, 2017, 2018, 2019a, b; Dayrat and Goulding 2017; Goulding et al. 2018a, b, c). Moreover, based on its known distribution (Andaman Islands, eastern Sumatra, western Peninsular Malaysia), *O. melakense* is expected to be found in other places, such as the Nicobar Islands. However, *O. melakense* is rare, at least in comparison to its two sympatric species, *O. typhae* and *O. stuxbergi*. Large populations (with dozens of individuals) of *O. typhae* were encountered (Dayrat et al. 2016). In the field, *O. typhae* and *O. stuxbergi* can be found by looking at the muddy surface of trunks, logs, and lobster mounds, while *O. melakense* can be found only if one actively searches for it under and inside logs.

##### Onchidium stuxbergi

Taxon classificationAnimaliaSystellommatophoraOnchidiidae

(Westerlund, 1883)

73DF9144-D2CC-53B6-A386-647A25FDDDDE

Figs 12B, C , 13C–E

Vaginulus stuxbergi Westerlund, 1883: 165; Westerlund 1885, 191–192, pl. 2, fig. 2a–c.
Onchidium stuxbergi (Westerlund, 1883): Dayrat et al. 2016: 21–32, figs 9–16.
Onchidium pallidipes Tapparone-Canefri, 1889: 329–331. Syn. nov.
Onchidium nigrum Plate, 1893: 188–190, pl. 8, fig. 31a, pl. 10, fig. 53, pl. 11, fig. 75; Hoffmann 1928: 78; Labbé 1934: 223–224, figs 58–61.
Elophilus ajuthiae Labbé, 1935: 312–317, figs 1–3. Elophilus Labbé, 1935, preoccupied by Elophilus Meigen, 1803 [Diptera], was replaced by Labbella Starobogatov, 1970.

###### Type material.

***Lectotype and paralectotypes*** (*Vaginulus stuxbergi*). Brunei DARUSSALAM • lectotype, 43/25 mm; Brunei Bay, northwestern Borneo; SMNH 1334. • 11 paralectotypes, 35/30 to 15/12 mm; SMNH 1334, SMNH 7523. For detailed information, see Dayrat et al. (2016: 22).

**Figure 12.**
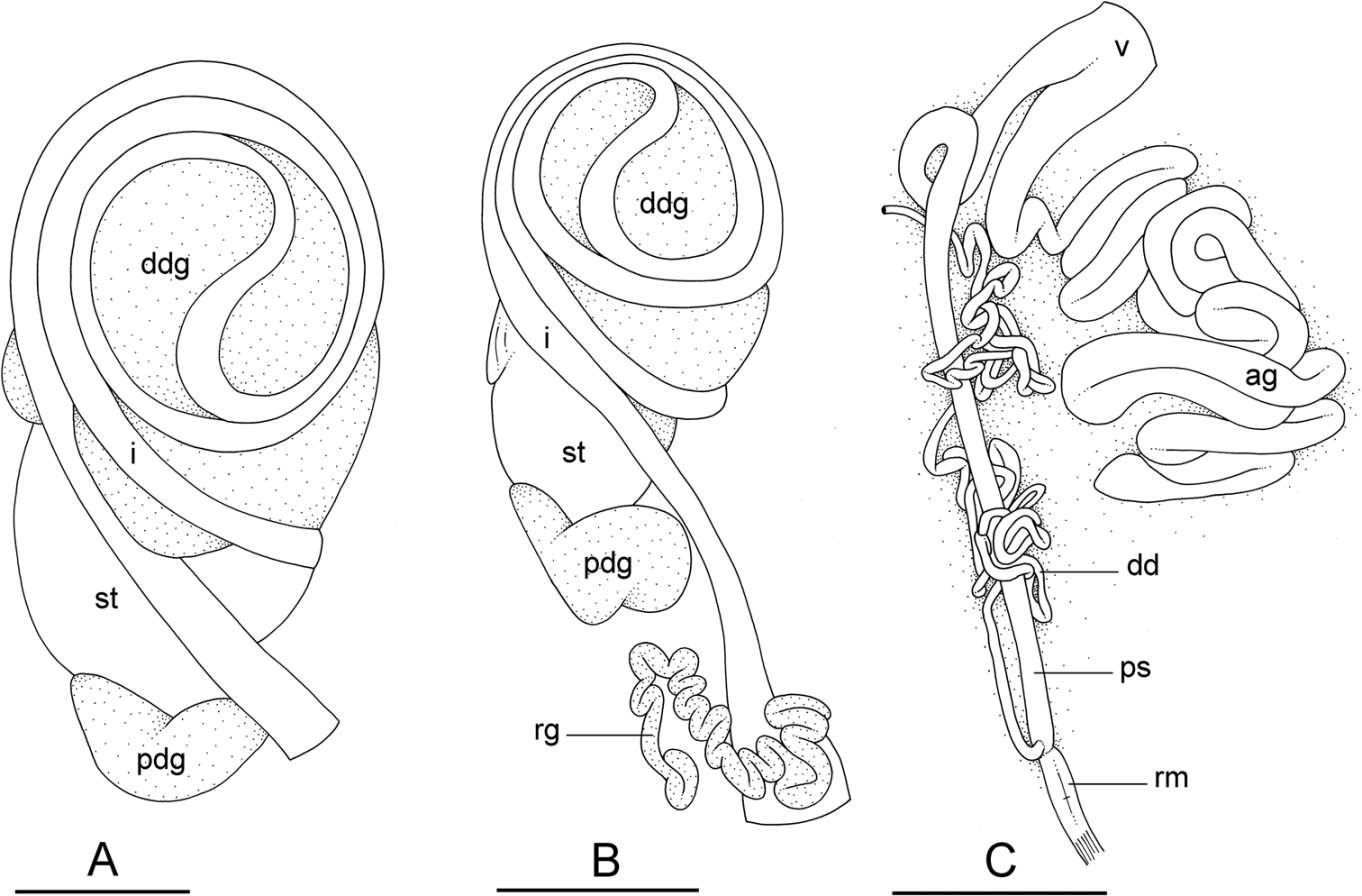
Name-bearing types of *Onchidium multinotatum* and *Onchidium pallidipes***A** digestive system, type III with a transitional loop at 2 o’clock (based on marks left by the intestine in the digestive gland), dorsal view, holotype, *O. multinotatum* (ZMB/Moll 240117) **B** digestive system, type III with a transitional loop at 2 o’clock, dorsal view, lectotype, *O. pallidipes* (NMNH 127328) **C** anterior, male, copulatory parts, lectotype, *O. pallidipes* (NMNH 127328). Abbreviations: ag accessory penial gland, dd deferent duct, ddg dorsal lobe of digestive gland, i intestine, pdg posterior lobe of the digestive gland, ps penial sheath, rg rectal gland, rm penial retractor muscle, st stomach, v vestibule. Scale bars: 3 mm (**A, B**), 2 mm (**C**).

***Lectotype and paralectotypes*** (*Onchidium pallidipes*). Myanmar • lectotype, 15/12 mm, designated here; Moulmein, Tenasserim [now Mawlamyine, Tanintharyi]; USNM 127328. • 1 paralectotype, 12/9 mm; same collection data as for the lectotype; ZMH 27467/1. • 1 paralectotype, 10/5 mm; same collection data as for the lectotype; ZMB/Moll 47190. The lectotype is poorly-preserved but its dorsal notum bears some faint traces of what could have been dorsal papillae similar to those found in *Onchidium*; its copulatory apparatus and its digestive system are drawn for the present study (Fig. 12B, C). One paralectotype is completely destroyed (ZMB/Moll 47190): it likely dried and it cannot be identified. The other paralectotype is an immature specimen with no male or female reproductive system (ZMH 27467/1), but its intestinal loops are exactly identical to those of the lectotype. Labels of the three type specimens indicate Moulmein as locality. All three type specimens seem to be from the same locality according to the original description (Tapparone-Canefri 1889: 330), and are preserved in three different museum collections.

***Holotype*** (*Onchidium nigrum*). Borneo • holotype, 40/30 mm, by monotypy; unidentified area on the island of Borneo; ZMB/Moll 22749. For detailed information, see Dayrat et al. (2016: 23).

***Syntypes*** (*Labbella ajuthiae*). Thailand • 3 syntypes 20/17 mm, 20/15 mm, and 20/14 mm; Chao Phraya River, Ayutthaya Province; brackish waters; MNHN-IM-2000-22965. For detailed information, see Dayrat et al. (2016: 23).

###### Additional material examined.

Indonesia – Sumatra • 1 specimen 23/14 mm [1775]; Dumai; 01°42.838'N, 101°23.286'E; 9 Oct 2012; M Khalil and field party leg.; st 74, mangrove forest just behind abandoned buildings, high intertidal, with many *Thalassina* mounds and small creeks; UMIZ 00003.

###### Distribution

(Fig. 4). Myanmar (type locality of *O. pallidipes*, new record), and eastern Sumatra (new record). Other known records are in Singapore, Sabah and western Peninsular Malaysia (Malaysia), Brunei Darussalam, Bohol (Philippines), Vietnam, Thailand (Gulf of Thailand), and southern China up to 22°10'N (Dayrat et al. 2016: 24).

###### Habitat.

In eastern Sumatra, *O. stuxbergi* was found on a muddy log, one of the habitats in which it is known to live (Dayrat et al. 2016: 24). In the original description of *O. pallidipes*, it is indicated that the slugs were found under the plant debris of sugar cane (Tapparone-Canefri 1889: 330), which is an unusual but possible habitat.

###### Abundance.

The present record from eastern Sumatra confirms that *O. stuxbergi* is not found in high densities (a few individuals at most) even though it is found at many sites across its distribution range.

###### Remarks.

Given its known records on the other side of the Strait of Malacca (western Peninsular Malaysia) and Singapore, *Onchidium stuxbergi* was expected to be present in eastern Sumatra. Anatomically, *Onchidium stuxbergi* in Sumatra is indistinguishable from the individuals found elsewhere. Also, the DNA sequences of the individual from eastern Sumatra are nested within the rest of the species (Fig. 1).

A detailed discussion on the synonymy of *Labbella ajuthiae* and *Onchidium nigrum* with *O. stuxbergi* can be found in Dayrat et al. (2016). The type material of *Onchidium pallidipes* was briefly addressed in a study on the genus *Melayonchis* Dayrat & Goulding in Dayrat et al. 2017. At the time, it was thought that *O. pallidipes* was a *nomen dubium*. However, the dissection of far more onchidiid species in the past few years has revealed that the coiled penial sheath of the lectotype of *O. pallidipes* (Fig. 12C) is typical of what is observed only in *Onchidium* (except for the new *Onchidium* species described here, in which the penial sheath is short and straight). Also, the poorly-preserved dorsal notum of the lectotype of *O. pallidipes* bears some faint traces of what could have been papillae similar to those found in *Onchidium*. So, now, it is considered that the name *Onchidium pallidipes* applies to an *Onchidium* species. Note that this application is exclusively based on the lectotype (designated here) because a paralectotype is destroyed and the other paralectotype is an immature specimen.

**Figure 13.**
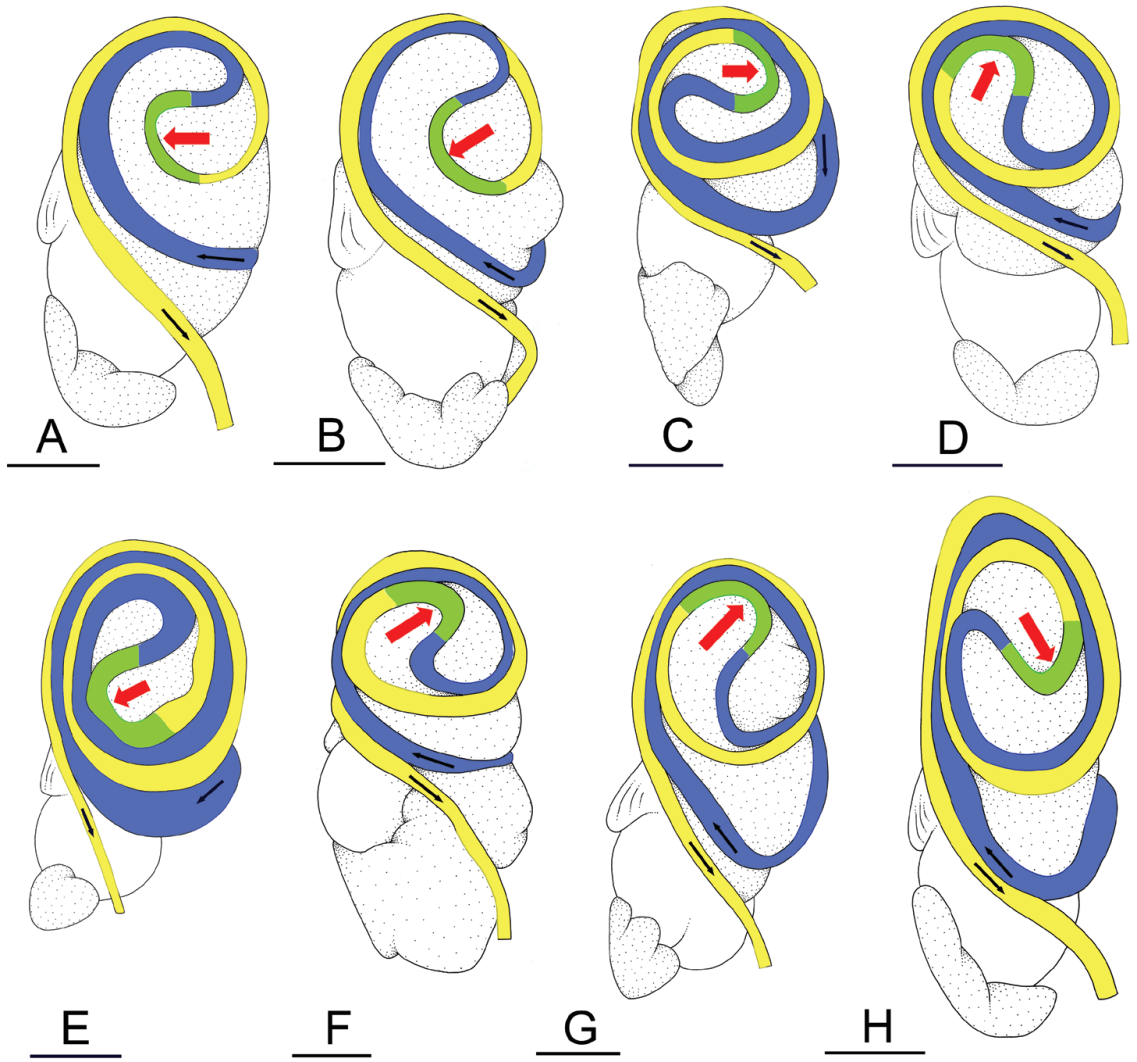
Types of intestinal loops in the genus *Onchidium*. Small black arrows indicate the direction of the intestinal transport, which starts in the blue loop. A blue loop turns clockwise. A yellow loop turns counterclockwise. A green loop is transitional in between a blue loop and a yellow loop. The orientation of the transitional (green) loop is indicated with a red arrow. Details on individuals of *O. reevesii*, *O. typhae*, *O. stuxbergi* can be found in Dayrat et al. (2016)**A***O. typhae*, type II with a transitional loop at 9 o’clock, [1007] **B***O. typhae*, type II with a transitional loop at 8 o’clock (from Dayrat et al. 2016: fig. 5E) **C***O. stuxbergi*, type III with a transitional loop at 3 o’clock (from Dayrat et al. 2016: fig. 11B) **D***O. stuxbergi*, type III with a transitional loop at 1 o’clock, (PNM 041200) **E***O. stuxbergi*, type III with a transitional loop at 8 o’clock, [5605] **F***O. reevesii*, holotype, type III with a transitional loop at 2 o’clock (from Dayrat et al. 2016: fig. 14A) **G***O. melakense*, type III with a transitional loop at 1 o’clock, holotype [5979] (USMMC 00075) **H***O. melakense*, type III with a transitional loop at 5 o’clock, [5978] (USMMC 00076). Scale bars: 3 mm (**A, H**), 5 mm (**B, C, E–G**), 4 mm (**D**).

*Onchidium typhae* is supposedly present in Myanmar because it is known from West Bengal eastward all the way to Singapore (Fig. 4). However, *O. pallidipes* cannot apply to *O. typhae* because the intestinal loops of *O. typhae* are always of type II (see below, Fig. 13A, B). Given the intestinal loops of type III of its lectotype (Fig. 12B), *O. pallidipes* applies to *O. stuxbergi*, also characterized by intestinal loops of type III (Fig. 13C–E). The hollow spine of the accessory penial gland of the lectotype of *O. pallidipes* is 2.7 mm long, which is slightly outside the range known so far in *O. stuxbergi* (0.5 to 2 mm), but that character is expected to vary. No additional retractor muscle fibers were found in the distal part of the male apparatus of the lectotype of *O. pallidipes* (Fig. 12C), even though they are known to be present in *O. stuxbergi* (Dayrat et al. 2016: fig. 11C). However, the lack of an anterior, retractor muscle in the lectotype of *O. pallidipes* can be explained by the fact that it is relatively small (15 mm long) and poorly-preserved. Also, this trait was found to vary in *O. melakense* and it is possible that it also varies in *O. stuxbergi*, especially among small individuals. Finally, it is worth pointing out that Tapparone-Canefri (1889: 330) selected the specific name *pallidipes* to refer to the “pale foot” of the preserved specimens he examined for the original description. Tapparone-Canefri (1889: 330) did not have access to information on live animals but he suggested that the foot was “probably ocher in living specimens,” which fits well with *O. stuxbergi* (of which the foot is bright orange).

In the future, if fresh material collected from the type locality of *O. pallidipes* is shown to form its own reciprocally-monophyletic unit using both mitochondrial and nuclear DNA sequences, and if it is shown to be anatomically fully compatible with the lectotype of *O. pallidipes* (especially regarding the length of the spine of the accessory penial gland), then *O. pallidipes* could become a valid name for a distinct *Onchidium* species endemic to the eastern Andaman Sea. This hypothesis cannot be completely ruled out at this stage. However, given the data currently available and all the reasons given above, we regard *O. pallidipes* as a junior synonym of *O. stuxbergi*.

The name *Onchidium multinotatum* Plate, 1893 needs to be briefly discussed. Its type locality is Cavite, Manila, in Luzon, Philippines. The original description is quite detailed, as often with Plate, but the holotype (30/15 mm), by monotypy (ZMB/Moll 240117), is very poorly preserved because it likely dried for a while. Only a few destroyed pieces of the digestive system remain in the empty body wall. Some of the features that Plate described could unfortunately not be checked (rectal gland present, accessory penial gland present, penial gland spine 4 mm long, penis 30 mm long). Plate separated the intestine from the digestive gland but described intestinal loops of type II. However, marks left by the intestine on the dorsal aspect of the digestive glands suggest that the intestinal loops of *O. multinotatum* were of type III (Fig. 12A). Given the critical uncertainty regarding the type of intestinal loops and that traits described by Plate cannot be checked, *Onchidium multinotatum* is regarded as a *nomen dubium*. *Onchidium multinotatum* could apply to *O. stuxbergi*, but that is not certain. We collected many onchidiids from the Philippines, including in Batangas, just south of Manila in Luzon, and the only species that could match the anatomy of *O. multinotatum* (acknowledging some uncertainty) is *O. stuxbergi*. Unfortunately, the type locality of *O. multinotatum* is in a part of Manila which is now completely developed and we could not collect onchidiids there.

### Identification key

A key based on external characters is provided to help identify *Onchidium* slugs in the field. Information on the color of live individuals of *O. typhae* and *O. stuxbergi* is from Dayrat et al. (2016). Information on the color of live individuals of *O. reevesii* is from Wang et al. (2018).

**Table:** 

1	The foot is bright orange	O. stuxbergi^*^
–	The foot is not bright orange	**2**
2	The hyponotum is pure white and the dorsum is light brown	O. melakense^**^
–	The hyponotum is not pure white and the dorsum is brown	**3**
3	The hyponotum is light grey or beige-white, the foot is whitish or light yellow, and the dorsum is brown	O. reevesii^***^
–	The hyponotum and the foot vary between greyish and yellowish, and sometimes even greenish, and the dorsum is brown	O. typhae^****^

### Discussion

*Onchidium* slugs can easily be identified in the field at the generic and specific levels. Indeed, all live *Onchidium* slugs are characterized by two external features that distinguish them from other onchidiids: large, conical, pointed papillae, and very long and thin ocular tentacles (easily up to 20 mm). Also, each *Onchidium* species is characterized by a distinct color and, even though *O. stuxbergi*, *O. typhae*, and *O. melakense* are sympatric, they cannot be confused (see the Identification key above, and Table 3). The only other genus in which species can be easily distinguished in the field is *Melayonchis* Dayrat & Goulding in Dayrat et al. 2017, but, in most other onchidiid genera, such as *Peronina*, *Wallaconchis*, or *Paromoionchis*, species are cryptic externally. This could suggest that *Onchidium* and *Melayonchis* species are relatively older and that there has been enough time for external differences to accumulate. Finally, the discovery of *O. melakense* suggests that additional, rare, endemic *Onchidium* species possibly still are unknown, especially in the region of the Strait of Malacca, which seems to be its center of highest diversity.

Our molecular phylogenetic analyses (Figs 1, 2) indicate that *O. stuxbergi* and *O. reevesii* are most closely related, which is supported by the fact that their hyponotum bears black dots (absent in *O. typhae* and *O. melakense*, Table 3). As of today, *O. stuxbergi* and *O. reevesii* do not overlap geographically even though they get very close in southern China (Fig. 4). Their speciation is possibly related to adaptation to warm (*O. stuxbergi*) and colder (*O. reevesii*) waters.

Our knowledge of *O. reevesii* is based on the re-description of the holotype (Dayrat et al. 2016: 32–35) as well as a recent re-description of fresh material by Wang et al. (2018). The latter study begs discussion here. The foot sole of *O. reevesii* is said to be “whitish or light yellow” within the body of the species description (Wang et al. 2018: 2). In the discussion, some individuals from Cixi City, Zhejiang Province, are also mentioned with a yellow foot (Wang et al. 2018: 6). It is unclear whether those specimens from Cixi City belong to *O. reevesii*. However, it cannot be excluded that the color of the foot sole of *O. reevesii* might vary from white to yellow, instead of light yellow. More importantly, according to Wang et al. (2018: 6): “On the basis of COI sequences we misidentified two distinct species as *Onchidium* ‘*struma*’ (Sun et al. 2014). These were *O. reevesii* and *O. hongkongense*.” That is incorrect: Dayrat et al. (2016: 35) demonstrated that Sun et al. (2014) applied the *nomen nudum Onchidium* ‘*struma*’ to *O. reevesii* and *O. stuxbergi*. Also, Dayrat et al. (2019a: 22) showed that *Onchidium hongkongense* Britton, 1984 is a junior synonym of *Paromoionchis tumidus* (Semper, 1880) and therefore does not apply to an *Onchidium* species. Finally, the individuals misidentified as *Paraoncidium reevesii* (J.E. Gray, 1850) by Sun et al. (2014) actually belong to *Paromoionchis tumidus* (Dayrat et al. 2019a: 44): the combination *Paraoncidium reevesii* is erroneous because the species described as *Onchidium reevesii* by J.E. Gray (1850) belongs to the genus *Onchidium*, based on the re-description of its holotype (Dayrat et al. 2016: 32–35), and also because *Paraoncidium* Labbé, 1934 is a junior synonym of *Onchidina* Semper, 1882 (Dayrat and Goulding 2017: 123).

In onchidiids, types of intestinal loops are defined based on the pattern of the intestine on the dorsal aspect of the digestive gland. Plate (1893) first distinguished four types of intestinal loops (types I to IV) and Labbé (1934) later added a type V. Only the types II and III are found in *Onchidium* (Table 3). The type species, *O. typhae*, is characterized by intestinal loops of type II, and the three other species are characterized by intestinal loops of type III. The different types of intestinal loops and their individual variation are best revealed by coloring with a different color different sections of the intestine (Dayrat et al. 2019b): a clockwise intestinal loop is colored in blue, a counterclockwise intestinal loop is colored in yellow, and a transitional loop between them is colored in green (Fig. 13).

The intestine first appears dorsally on the right side and starts by forming a clockwise (blue) loop (Fig. 13). In intestinal loops of type II, the clockwise (blue) loop makes approximately a complete circle. As a result, the transitional (green) loop is oriented to the left, typically at 9 o’clock (horizontal red arrow, Fig. 13A). In *Onchidium*, intestinal loops of type II are found only in *O. typhae*, in which the orientation of the transition loop varies approximately between 8 and 9 o’clock (Fig. 13A, B). In intestinal loops of type III, the clockwise (blue) loop is longer and rotates more than in a type II. As a result, the transitional (green) loop is oriented to the right, typically at 3 o’clock (horizontal red arrow, Fig. 13C). In *O. stuxbergi*, the orientation of the transitional (green) loop varies from 1 to 8 o’clock (red arrow, Fig. 13C–E). In *O. reevesii*, the orientation of the transitional (green) loop is approximately at 2 o’clock (red arrow, Fig. 13F). In *O. melakense*, the orientation of the transitional (green) loop varies from 1 to 5 o’clock (red arrow, Fig. 13G, H). A few preliminary remarks on the distribution of types of intestinal loops in genera of onchidiid slugs can be found in Dayrat et al. (2019b). A more thorough discussion regarding types of intestinal loops will be provided after our revisions of *Peronia* and *Platevindex* are published (in preparation).

## Supplementary Material

XML Treatment for Onchidium

XML Treatment for Onchidium melakense

XML Treatment for Onchidium stuxbergi

## Footnotes

Known distribution: Myanmar, western Peninsular Malaysia, eastern Sumatra, Thailand (Gulf of Thailand), Vietnam, eastern Borneo, Philippines, and southernmost tropical China (up to 22°10'N).

Known distribution: Andaman Islands, western Peninsular Malaysia, and eastern Sumatra.

Known distribution: subtropical China, from 22°30'N to 34°36'N.

Known distribution: West Bengal, Andaman Islands, western Peninsular Malaysia, and Singapore.
